# Electrochemical/Colorimetric Dual-Mode Aptasensor Based on CuZr-MOF and Fe_3_O_4_@ZIF-8 for Detection of Malathion in Vegetables

**DOI:** 10.3390/bios16020101

**Published:** 2026-02-04

**Authors:** Kaili Liu, Jiwei Dong, Youkai Wang, Jiashuai Sun, Peisen Li, Yemin Guo, Xia Sun

**Affiliations:** 1 School of Agricultural Engineering and Food Science, Shandong University of Technology, No. 266 Xincun West Road, Zibo 255049, China; mykaili0905@gmail.com (K.L.); xiaodongzainuli@163.com (J.D.); 13791826843@163.com (Y.W.); sunjiashuai97@163.com (J.S.); lipeisen1999@163.com (P.L.); 2 Shandong Muyang New Energy Co., Ltd., Fulai Industrial Park, Rizhao 276800, China; 3 Shandong Provincial Engineering Research Center of Vegetable Safety and Quality Traceability, No. 266 Xincun West Road, Zibo 255049, China

**Keywords:** electrochemical/colorimetric dual-mode, magnetic nanoparticle, on-site testing

## Abstract

In on-site rapid detection, the electrochemical method boasts high sensitivity and rapid response capabilities, while the colorimetric method can provide intuitive visual readings suitable for on-site screening. Therefore, this study developed an innovative dual-mode electrochemical/colorimetric aptasensor for the accurate detection of malathion (MAL) in vegetables. The sensor combines magnetic Fe_3_O_4_@ZIF-8-DNA composites and CuZr-MOF-cDNA probes, enabling simultaneous detection of the target through electrochemical reactions and colorimetric changes. The introduction of CuZr-MOF not only enhances the sensor’s conductivity but also significantly amplifies the electrochemical signal through its catalytic properties. The magnetic Fe_3_O_4_@ZIF-8-DNA composite facilitates solid–liquid separation under an external magnetic field. When the target MAL is present, the aptamer binds to the target, causing the CuZr-MOF-cDNA probes to release from the composite, altering the number of free probes in the supernatant and generating varying intensities of colorimetric signals. Meanwhile, the MAL captured in the precipitate by the aptamer is quantitatively detected through electrochemical methods. Experimental results demonstrate that as the target concentration increases, the colorimetric signal intensifies while the electrochemical signal weakens, showing a good linear relationship between the two. The aptasensor’s limit of detection (LOD) for colorimetric and electrochemical modes was 1.57 × 10^−11^ M and 4.76 × 10^−11^ M, respectively, with recoveries ranging from 87.71% to 107.68% and relative standard deviations between 3.23% and 10.75%. This method exhibits high sensitivity, excellent selectivity, and strong reliability, providing a novel technique for the accurate quantification of MAL in vegetables, particularly suited for on-site rapid detection.

## 1. Introduction

In recent years, with the intensification of agricultural practices, organophosphorus pesticides (OPs) have been widely used in vegetable cultivation due to their high efficiency and broad-spectrum insecticidal properties [1]. However, the excessive or improper use of OPs has led to significant residue issues, posing serious threats to food safety and human health. Among them, MAL, a typical organophosphorus pesticide, exerts neurotoxic effects by inhibiting acetylcholinesterase activity, and long-term exposure to low doses may lead to chronic diseases [2]. Although conventional detection techniques such as chromatography and mass spectrometry offer high accuracy, they rely on sophisticated instruments and trained personnel, making them unsuitable for rapid on-site detection [3]. Therefore, the development of highly sensitive, easy-to-operate, and field-deployable detection methods has become a critical focus in current research [4].

Electrochemical biosensors have garnered increasing attention in pesticide detection due to their rapid response, high sensitivity, and potential for miniaturization [5,6,7]. However, single-mode electrochemical sensors are susceptible to environmental interferences, which may result in false-positive or false-negative outcomes [8]. In contrast, colorimetric assays offer intuitive visual readouts based on observable color changes, making them suitable for rapid on-site screening. Nonetheless, their quantification accuracy is often limited by subjective interpretation of color intensity [9]. The integration of both techniques into a dual-mode sensor allows for cross-validation of results, thereby significantly enhancing the accuracy and reliability of detection [10]. Aptamers, known for their high specificity and strong binding affinity, serve as ideal molecular recognition elements for dual-mode sensor design [11].

Based on this concept, this study proposes an electrochemical/colorimetric dual-mode aptasensor for the accurate detection of MAL in vegetables, utilizing Copper-zirconium metal–organic framework (CuZr-MOF) and magnetic nanoparticles (Fe_3_O_4_@ZIF-8) as core functional materials. The innovation of this sensor lies in several aspects: first, the magnetic Fe_3_O_4_@ZIF-8 nanocomposite enables rapid magnetic separation, simplifying the sample pretreatment process; second, CuZr-MOF serves as a signal amplification carrier, whose high surface area and abundant metal active sites significantly enhance the electrochemical response while catalyzing colorimetric reactions to boost colorimetric signal intensity. Through a competitive binding mechanism between the aptamer and its complementary DNA (cDNA), the sensor can release the probe in the presence of MAL, generating dual responses—an electrochemical signal in the precipitate phase and a colorimetric signal in the supernatant. The inverse relationship between these signals provides cross-verification, effectively reducing the risk of false positives. Furthermore, by integrating the signal amplification effects of nanomaterials and the synergistic advantages of dual-mode detection, the sensor exhibits excellent sensitivity and anti-interference capability. The aptasensor’s limit of detection (LOD) for colorimetric and electrochemical modes was 1.57 × 10^−11^ M and 4.76 × 10^−11^ M, respectively, with recoveries ranging from 87.71% to 107.68% and relative standard deviations between 3.23% and 10.75%. This study not only offers a novel and reliable technique for detecting OP residues but also opens new avenues for rapid on-site detection of trace contaminants in complex matrices, with significant implications for food safety and the advancement of portable detection devices.

## 2. Supplementary Note

The preparation of materials is provided in the Supporting Information.

## 3. Results and Discussion

### 3.1. Construction and Detection Principle of the Electrochemical/Colorimetric Dual-Mode Aptasensor

**Figure 1:**
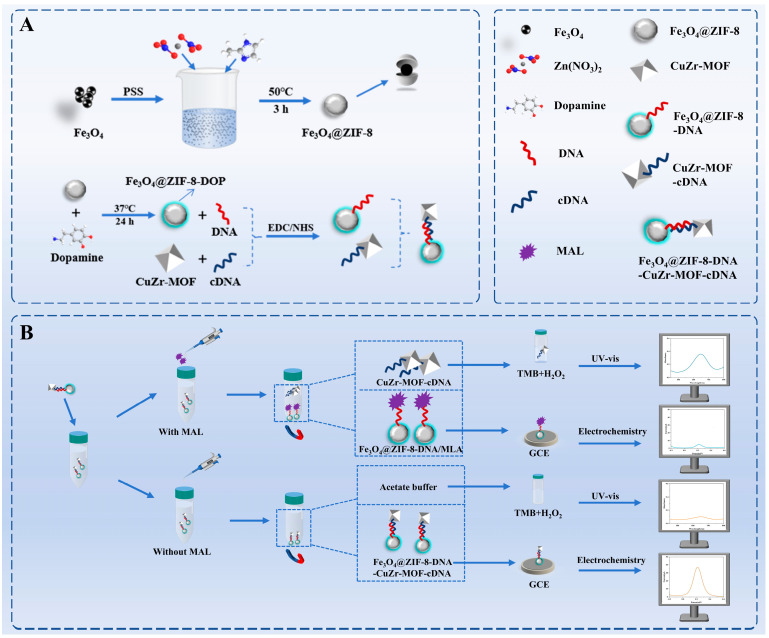
(**A**) Synthesis of Nanomaterials; (**B**) Detection Schematic Diagram of the Dual-Mode Sensor.

As shown in Figure 1A, magnetic nanocomposite materials Fe_3_O_4_@ZIF-8 and bimetallic CuZr-MOF were prepared. The prepared magnetic nanocomposite Fe_3_O_4_@ZIF-8 was used to fix the carboxyl-modified aptamer through amide bonds, resulting in Fe_3_O_4_@ZIF-8-DNA. The amino-modified cDNA was covalently immobilized onto the carboxyl-rich CuZr-MOF (synthesized with the H_2_BDC ligand) via the EDC/NHS chemistry method, yielding CuZr-MOF-cDNA. The two composites were assembled through the specific binding between the aptamer DNA and complementary chain cDNA.

As shown in Figure 1B, in the initial state, a large number of CuZr-MOF-cDNA probes were fixed on the Fe_3_O_4_@ZIF-8-DNA composite material, with almost no free CuZr-MOF-cDNA probes in the supernatant. When the target compound, MAL, was introduced, the aptamer preferentially bound to the target, leading to the release of CuZr-MOF-cDNA probes from the Fe_3_O_4_@ZIF-8-DNA composite material. Some probes entered the supernatant. Through magnetic separation, the precipitate and supernatant were separated. The precipitate retained the un-released CuZr-MOF-cDNA probes, which were detected electrochemically. The electrochemical response stems from the redox reaction of the [Fe(CN)_6_]3-/4-probe. Its signal attenuation is attributed to the compromised electron transfer, resulting from the formation of the aptamer–target complex and the release of conductive CuZr-MOF from the electrode surface. The free CuZr-MOF-cDNA probes in the supernatant participated in the 3,3′,5,5′-Tetramethylbenzidine (TMB)-H_2_O_2_ catalytic reaction, producing a blue oxidation product, and the colorimetric signal was measured using a UV–visible spectrophotometer. This colorimetric signal is generated because the released CuZr-MOF acts as a nanozyme, catalyzing the H_2_O_2_-mediated oxidation of TMB to blue oxTMB.

As the concentration of the target compound increases, more CuZr-MOF-cDNA probes are released from the composite material, causing the colorimetric signal to gradually increase. At the same time, due to the release of CuZr-MOF and the capture of aptamers that hinder electron transfer, the electrochemical signal gradually decreases. This “dual-mode differential response” characteristic enables cross-validation of the signals, enhancing the accuracy and reliability of the detection system.

### 3.2. Characterization of Nanomaterials

**Figure 2:**
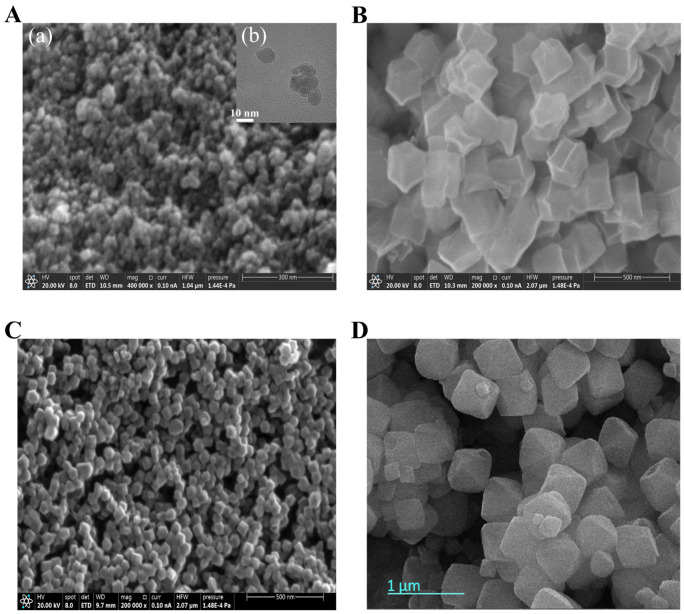
(**A**) SEM image (**a**), TEM image (**b**) of Fe_3_O_4_; (**B**) SEM image of ZIF-8; (**C**) SEM image of Fe_3_O_4_@ ZIF-8; (**D**) SEM image of CuZr-MOF.

The synthesized nanomaterials, including Fe_3_O_4_, ZIF-8, and Fe_3_O_4_@ZIF-8, were systematically characterized by scanning electron microscopy (SEM) and transmission electron microscopy (TEM), as shown in Figure 2. As illustrated in the SEM image of Fe_3_O_4_ (Figure 2A(a)), the particles exhibit a uniform quasi-spherical morphology. The corresponding TEM image (Figure 2A(b)) reveals an average particle size of approximately 10 nm.

For ZIF-8 (Figure 2B), the SEM image shows a typical rhombic dodecahedron shape with an average particle size of about 300 nm. The smooth surface and well-defined crystal facets are consistent with the anisotropic growth characteristics of metal–organic frameworks (MOFs) regulated by 2-methylimidazole. In contrast, the SEM image of the Fe_3_O_4_@ZIF-8 composite (Figure 2C) indicates a smaller particle size of approximately 100 nm, which is about 70% smaller than that of pure ZIF-8. This size reduction is closely related to the altered nucleation dynamics induced by the core–shell structure. According to classical crystal growth theory, the Fe_3_O_4_ nanocores act as heterogeneous nucleation sites, significantly reducing the activation energy for the growth of the ZIF-8 shell and thereby promoting the formation of smaller crystals.

Furthermore, no free Fe_3_O_4_ nanoparticles were observed on the surface of the composite, which can be attributed to the surface functionalization strategy employed during synthesis. The Fe_3_O_4_ nanoparticles were pretreated with sodium polystyrene sulfonate (PSS), which enhanced the surface negative charge density and ensured a more uniform charge distribution. This facilitated the uniform and continuous growth of the ZIF-8 shell, with an average thickness of approximately 100 nm, and promoted the formation of ZIF-8 crystal nuclei.

These results indicate that the synthesized Fe_3_O_4_@ZIF-8 composites possess the desired core–shell structure and meet the design expectations.

The SEM image of CuZr-MOF is presented in Figure 2D. Detailed characterization and validation of CuZr-MOF have been reported in our previous work [12].

**Figure 3:**
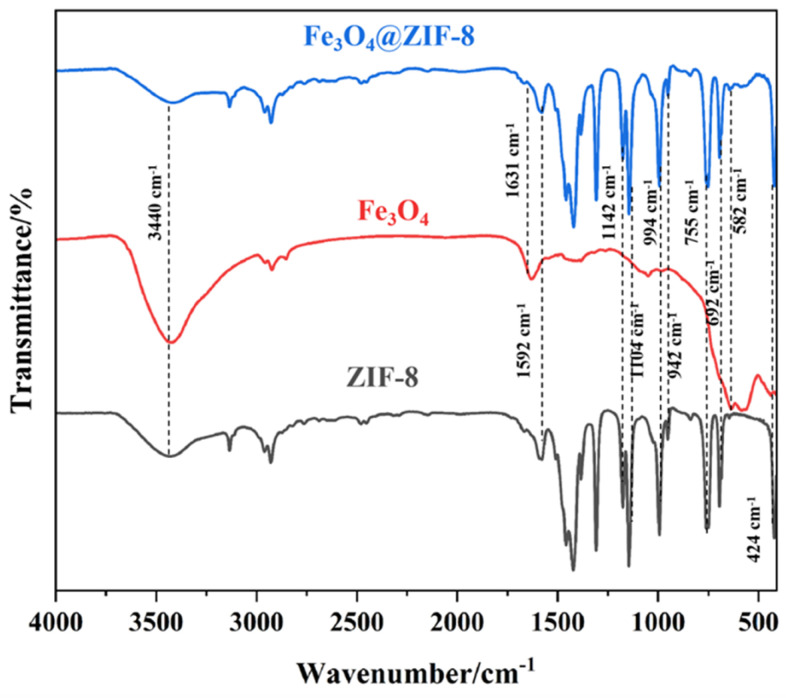
FT-IR image of Fe_3_O_4_@ ZIF-8, Fe_3_O_4_ and ZIF-8.

To verify the successful synthesis of the Fe_3_O_4_@ZIF-8 composite, Fourier transform infrared spectroscopy (FTIR) was employed to comparatively analyze Fe_3_O_4_, ZIF-8, and the composite material (Figure 3), with key spectral bands assigned and interpreted. In the spectrum of Fe_3_O_4_@ZIF-8, a strong absorption peak at 582 cm^−1^ corresponds to the stretching vibration of the Fe–O bond, which closely matches the characteristic peak of pure Fe_3_O_4_ at 581 cm^−1^, confirming the preservation of the magnetic core. A distinct vibration band at 424 cm^−1^ is attributed to the Zn-N coordination bond, indicative of the coordination interaction between the metal nodes and 2-methylimidazole ligands in ZIF-8, thereby verifying the successful formation of the ZIF-8 framework.

Within the 500–1500 cm^−1^ region, characteristic bands associated with the imidazole ring vibrations are observed: peaks at 755 cm^−1^ and 692 cm^−1^ correspond to out-of-plane bending vibrations, those at 994 cm^−1^ and 1145 cm^−1^ relate to in-plane C–H bending, and the peak at 1308 cm^−1^ is attributed to C-N stretching. A sharp peak at 1592 cm^−1^ arises from symmetric stretching of the C=N bond in the imidazole ring, while the broad band at 3440 cm^−1^ is associated with C-H stretching vibrations from the methyl group of the ligand.

These results confirm that Fe_3_O_4_@ZIF-8 possesses the characteristic functional groups of both Fe_3_O_4_ and ZIF-8, demonstrating the formation of a core–shell structure. Furthermore, the shift in peak positions is less than 5 cm^−1^, indicating that the composite process does not alter the intrinsic chemical structures of the individual components.

**Figure 4:**
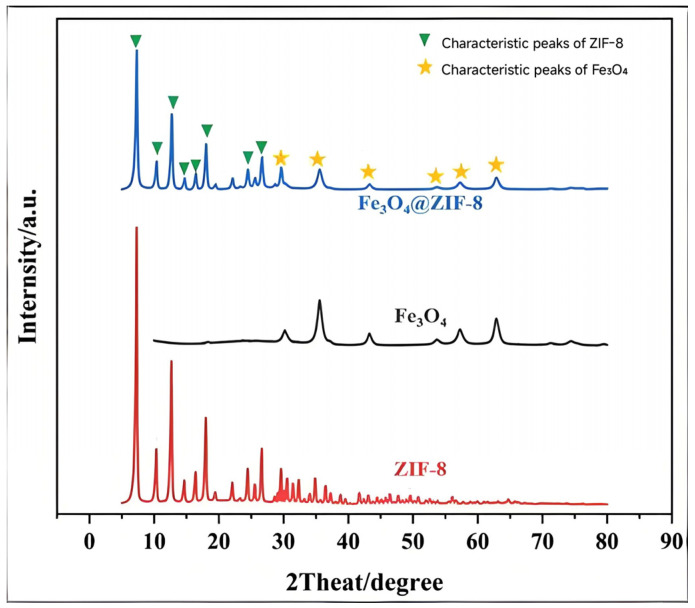
XRD images of Fe_3_O_4_@ ZIF-8, Fe_3_O_4_ and ZIF-8.

To investigate the crystal structures of the synthesized Fe_3_O_4_ nanoparticles, ZIF-8, and the Fe_3_O_4_@ZIF-8 composite, X-ray diffraction (XRD) analysis was performed. As shown in Figure 4, six distinct diffraction peaks were observed in the XRD pattern of Fe_3_O_4_ nanoparticles at 2θ values of 29.7°, 35.0°, 42.5°, 52.8°, 56.6°, and 62.4°, corresponding to the (220), (311), (400), (422), (511), and (440) crystal planes, respectively. These peaks are in good agreement with the typical diffraction pattern of Fe_3_O_4_ reported in the literature [13], confirming the high crystallinity of the synthesized Fe_3_O_4_.

For ZIF-8, the XRD pattern exhibits a series of characteristic diffraction peaks at 7.3°, 10.3°, 12.7°, 14.7°, 16.4°, 18.0°, 24.6°, and 26.7°, which can be indexed to the (011), (002), (112), (022), (013), (222), (233), and (134) planes of ZIF-8, respectively. These results are consistent with the reported diffraction features of ZIF-8 in the literature [14], indicating good crystallinity of the synthesized ZIF-8 material.

In the XRD pattern of the Fe_3_O_4_@ZIF-8 composite, diffraction peaks corresponding to both Fe_3_O_4_ and ZIF-8 are observed, confirming that the ZIF-8 framework has been successfully coated onto the Fe_3_O_4_ nanoparticles and that the crystal structures of both components coexist. This indicates that the introduction of ZIF-8 does not alter the intrinsic crystal structure of Fe_3_O_4_ nanoparticles. Moreover, the retention of the characteristic peaks of ZIF-8 suggests that the structural integrity of the framework was preserved during the composite formation.

The XRD analysis also implies potential interactions between the Fe_3_O_4_ nanoparticles and the ZIF-8 crystals, which may facilitate the growth of the ZIF-8 framework on the surface of Fe_3_O_4_ via heterogeneous nucleation.

**Figure 5:**
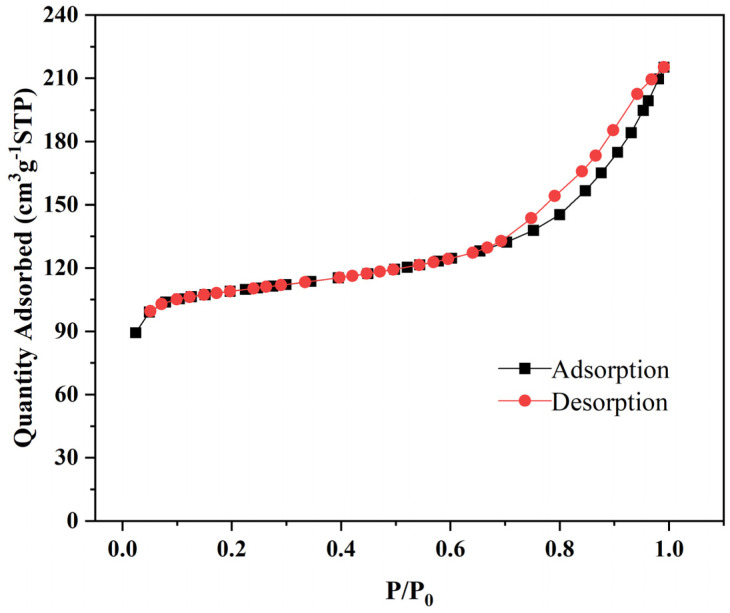
N_2_ adsorption and desorption image of Fe_3_O_4_@ZIF-8.

Figure 5 shows the nitrogen adsorption–desorption isotherm of Fe_3_O_4_@ZIF-8, which exhibits a typical type I isotherm. This is indicative of the rapid filling of micropores with diameters ranging from 1 to 1.5 nm, characteristic of the ZIF-8 structure. After the initial adsorption phase, the isotherm becomes nearly horizontal, suggesting minimal additional adsorption once the micropores are saturated. At higher relative pressures (P/P_0_ > 0.7), a noticeable hysteresis loop is observed between the adsorption and desorption branches, which is attributed to the interparticle voids formed by the aggregation of Fe_3_O_4_@ZIF-8 particles. The specific surface area of Fe_3_O_4_@ZIF-8 is measured to be 331.3 m^2^/g, which is significantly lower than the reported value for pure ZIF-8 (1173 m^2^/g). This reduction is due to the presence of the Fe_3_O_4_ core, which contributes little to the surface area, with the majority of the specific surface area originating from the ZIF-8 shell.

### 3.3. Feasibility Verification of the Dual-Mode Sensor

#### 3.3.1. Verification of Peroxidase-like Activity

To systematically evaluate the peroxidase-like catalytic activity of CuZr-MOF, the classical TMB-H_2_O_2_ colorimetric assay was employed [15]. In the presence of CuZr-MOF nanozyme, a characteristic absorption spectrum corresponding to oxidized TMB (oxTMB) was observed in the TMB and hydrogen peroxide mixture, along with a distinct blue coloration.

**Figure 6:**
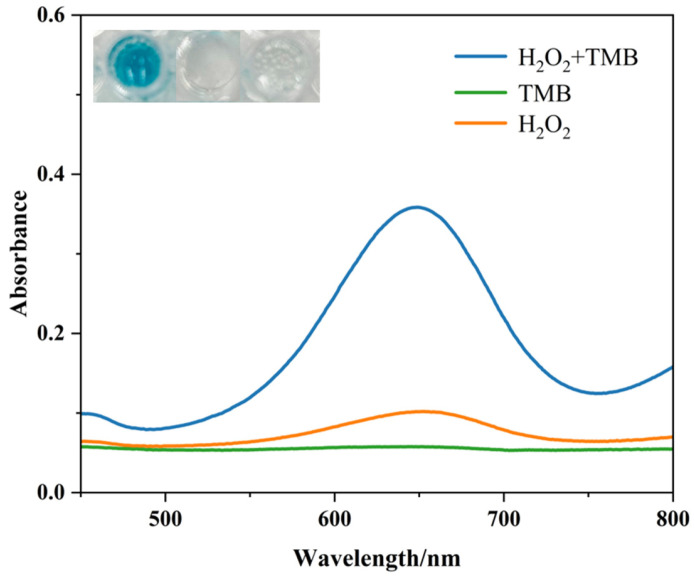
Validation of the catalytic activity of CuZr-MOF-like catalase by colorimetric assay.

In contrast, when CuZr-MOF was added to solutions containing only H_2_O_2_ or only TMB, no significant color change or absorbance increase was detected (Figure 6). These results clearly indicate that the formation of oxTMB originates from the intrinsic peroxidase-like activity of CuZr-MOF. In the presence of H_2_O_2_, CuZr-MOF catalyzes the oxidation of TMB, producing oxTMB with a characteristic absorption signal.

#### 3.3.2. Electrochemical Characterization

**Figure 7:**
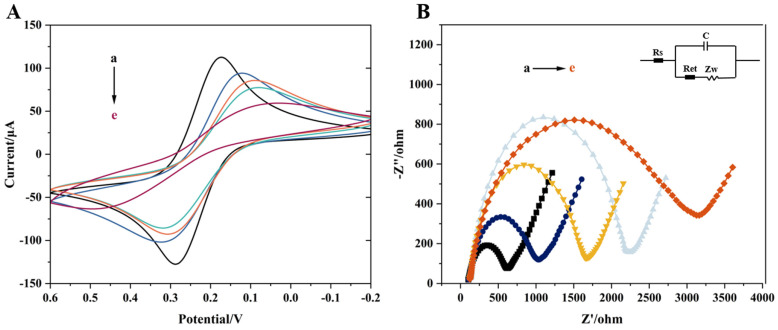
Changes in (**A**) CV and (**B**) EIS of the electrode surfaces during modification of the electrodes in a 5 mM [Fe(CN)_6_]^3−/4−^ solution containing 0.1 M KCl of (a) GCE, (b) GCE/Fe_3_O_4_@ZIF-8, (c) GCE/Fe_3_O_4_@ZIF-8-DNA@cDNA-CuZr-MOF, (d) GCE/Fe_3_O_4_@ZIF-8-DNA, and (e) GCE/Fe_3_O_4_@ZIF-8-DNA@cDNA-CuZr-MOF/MAL.

To verify the electrochemical response of different modification materials, cyclic voltammetry (CV) and electrochemical impedance spectroscopy (EIS) were used to evaluate the modification process of the electrode with different materials. As shown in Figure 7, curves a to e correspond to an unmodified GCE, GCE/Fe_3_O_4_@ZIF-8, GCE/Fe_3_O_4_@ZIF-8-DNA@cDNA-CuZr-MOF, GCE/Fe_3_O_4_@ZIF-8-DNA, and GCE/Fe_3_O_4_@ZIF-8-DNA@cDNA-CuZr-MOF/MAL, respectively.

In Figure 7A, the bare GCE exhibits clear redox peaks, indicating a good electrochemical signal. After modification with the synthesized nanocomposite Fe_3_O_4_@ZIF-8, due to the poor conductivity of Fe_3_O_4_@ZIF-8, the redox peaks decrease, and the peak separation (ΔEp) increases. When the aptamer is attached to Fe_3_O_4_@ZIF-8 to form GCE/Fe_3_O_4_@ZIF-8-DNA, the redox peaks further decrease, and the ΔEp value increases significantly. This is because the negatively charged phosphate groups in the aptamer chain are repelled by the similarly negatively charged [Fe(CN)_6_]^3−/4−^ ions in the electrolyte, hindering the electron transfer at the electrode surface. After the specific binding of CuZr-MOF with the complementary chain cDNA, the electrode surface forms GCE/Fe_3_O_4_@ZIF-8-DNA@cDNA-CuZr-MOF, and the redox peaks increase. This phenomenon occurs because CuZr-MOF influences the electrochemical signal, although the binding between the aptamer and its complementary chain limits the enhancement effect of CuZr-MOF on the electrochemical signal. Finally, in the presence of the target pesticide MAL, the specific binding of the target with cDNA inhibits the binding of DNA and cDNA, leading to a significant decrease in the redox peak and a significant increase in ΔEp. This change reflects not only the hindrance of electron transfer caused by the aptamer–organophosphorus pesticide complex formation but also the dissociation of the conductive CuZr-MOF with cDNA.

Figure 7B shows the EIS curves, further reflecting the characteristics of the electrode interface during the electrochemical detection process. From curve a to curve e, the semicircle diameter gradually increases, indicating a gradual increase in electron transfer resistance. This change is consistent with the analysis of the CV curves and validates the effect of different modification materials on the electrochemical response of the electrode.

By comparing curves c and e, the feasibility of the dual-mode aptasensor for electrochemical detection can be further confirmed. Curve c represents the electrochemical signal in the target solution without the presence of the target MAL, while curve e represents the electrochemical signal when the target MAL is present. The results show that the electrochemical signal significantly decreases in the presence of the target, which corresponds to the signal suppression effect caused by the aptamer–target binding.

### 3.4. Optimization of Experimental Conditions

In the construction of the electrochemical/colorimetric dual-mode sensor, several key factors—namely the concentration of nanomaterials, the concentration of aptamers, the incubation time between aptamers and targets, the pH of the electrochemical test buffer, and the pH of the acetate buffer used in colorimetric detection—significantly affect the performance of the sensor. Therefore, optimization experiments were carried out for these six parameters.

**Figure 8:**
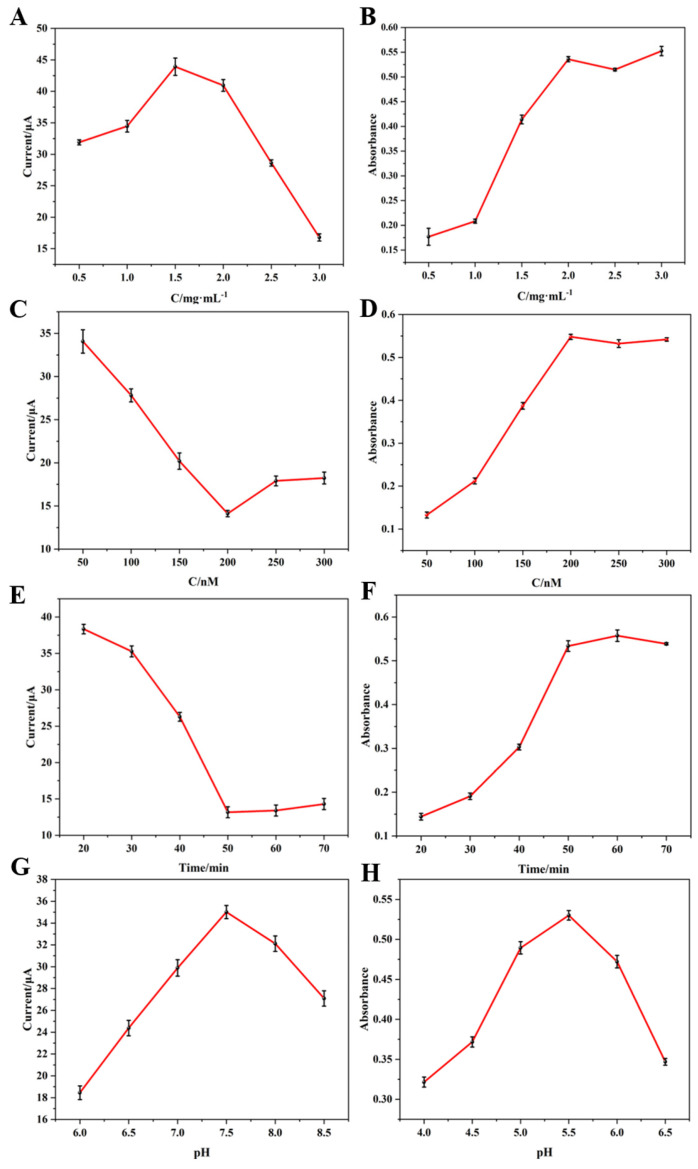
Optimization of experimental conditions: (**A**,**B**) optimization of nanomaterial concentration; (**C**,**D**) optimization of aptamer concentration; (**E**,**F**) optimization of incubation time for aptamer and target; (**G**) optimization of [Fe(CN)_6_]^3−/4−^ pH for electrochemical testing of the substrate; and (**H**) optimization of pH of sodium acetate buffer.

The concentration of the nanocomposite material influences both electrochemical and colorimetric detection. Optimization was performed using different concentrations of Fe_3_O_4_@ZIF-8-DNA@cDNA-CuZr-MOF (with a Fe_3_O_4_@ZIF-8-DNA to cDNA-CuZr-MOF ratio of 1:1, aptamer concentration of 200 nM, and testing buffer at pH 7.5). As shown in Figure 8A, the electrochemical signal increased with increasing concentration of nanomaterials; however, when the concentration increased from 1.5 mg/mL to 2 mg/mL, the signal slightly declined, and it dropped sharply at higher concentrations. This is attributed to the excessive thickness of the nanomaterial layer on the electrode surface, which increases the electron transfer path and interfacial impedance. In colorimetric detection (Figure 8B), the supernatant obtained after adding the target (1 μM) was used for the TMB reaction. At low concentrations of nanomaterials, the absorbance of TMB oxidation in the supernatant was low. As the concentration increased, the absorbance increased until stabilizing at 2 mg/mL. Based on both electrochemical and colorimetric results, 2 mg/mL was selected as the optimal concentration.

The aptamer concentration directly affects target recognition and influences the specific hybridization between nanomaterials via aptamer and cDNA. Under the optimal nanomaterial concentration, different concentrations of aptamers (DNA and cDNA at a 1:1 ratio) were incubated with 1 μM MAL for 50 min. The supernatant and precipitate were separately analyzed by electrochemical and colorimetric methods. As shown in Figure 8C, the electrochemical signal decreased as the aptamer concentration increased, reaching a minimum at 200 nM. Increasing the concentration further to 250 nM caused a slight increase in the signal, which then leveled off. This is because at low aptamer concentrations, the binding efficiency with the target is insufficient and CuZr-MOF and Fe_3_O_4_@ZIF-8 are not fully hybridized, so the precipitate is mainly magnetic Fe_3_O_4_@ZIF-8. As the aptamer concentration increases, more target–aptamer complexes form, reducing the signal. When saturation is reached at 200 nM, excess CuZr-MOF in the precipitate increases the signal slightly. Figure 8D shows consistent results: after 200 nM, the absorbance no longer increases. Therefore, 200 nM was selected as the optimal aptamer concentration.

To ensure complete binding between aptamer and target, the incubation time with 1 μM target was optimized under the above conditions. As shown in Figure 8E,F, at 50 min, the electrochemical signal reached a minimum and the colorimetric signal reached a maximum, after which the signal plateaued. To ensure full binding while minimizing non-specific interactions, 60 min was chosen as the optimal incubation time.

The pH of the electrochemical test solution was optimized as shown in Figure 8G. The electrochemical signal peaked at pH 7.5, so a slightly alkaline pH of 7.5 was selected for the test buffer. Lastly, the pH of the acetate buffer for the H_2_O_2_-mediated TMB reaction was optimized. As shown in Figure 8H, the absorbance increased steadily from pH 4.0 to 5.5, then decreased from pH 5.5 to 6.5. Thus, pH 5.5 was chosen as the optimal pH for the acetate buffer in the colorimetric assay.

Optimized experimental conditions: concentration of nanocomposite 2 mg/mL, concentration of aptamer 200 nM, incubation time of aptamer with target 60 min, pH of the supporting electrolyte for electrochemical tests 7.5, pH of sodium acetate buffer 5.5.

### 3.5. Performance Analysis of the Dual-Mode Aptasensor

**Figure 9:**
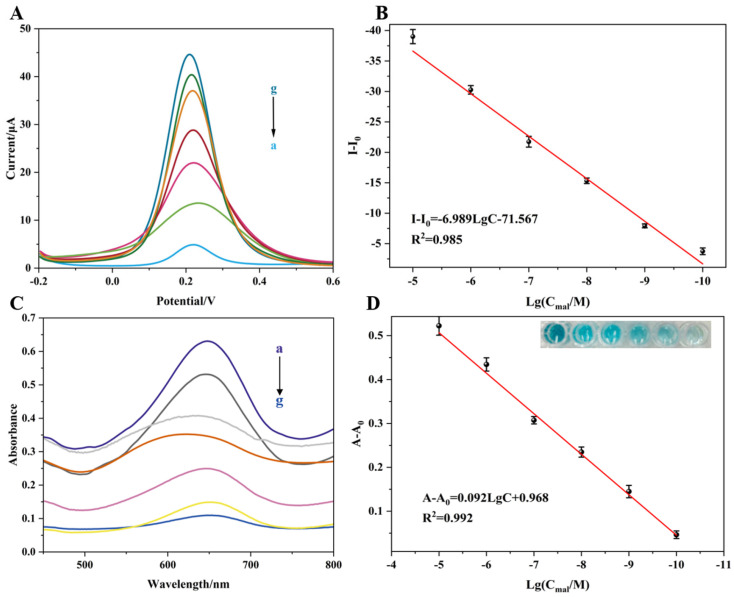
Detection of MAL at different concentration gradients by the prepared electrochemical/colorimetric dual-mode aptasensor. (**A**) DPV response of different concentrations of MAL; (**B**) electrochemical standard curve of the aptasensor for the detection of different concentrations of MAL; (**C**) UV-Vis absorption spectra of different concentrations of MAL; (**D**) colorimetric standard curve of the aptasensor for the detection of different concentrations of MAL. (a) 10^−5^ M; (b) 10^−6^ M; (c) 10^−7^ M; (d) 10^−8^ M; (e) 10^−9^ M; (f) 10^−10^ M; (g) 0 M.

**Figure 10:**
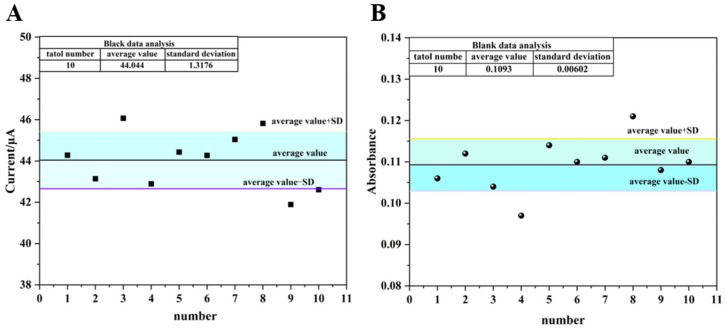
(**A**) Response of blank sample for electrochemical detection; (**B**) response of blank sample for colorimetric detection.

After optimizing the experimental conditions, the detection performance of the electrochemical/colorimetric dual-mode aptasensor for different concentrations of MAL was analyzed. As shown in Figure 9A, the differential pulse voltammetry (DPV) responses decreased progressively with increasing concentrations of MAL in the range from 10^−10^ to 10^−5^ M. Figure 9B illustrates the linear relationship between the logarithm of MAL concentration and the change in current response (I − I_0_, where I is the measured current response and I_0_ is the blank control). The fitted linear regression equation is:

I − I_0_ = −6.989 LgC − 71.567 (1)

with a correlation coefficient of R^2^ = 0.985. As shown in Figure 10A, ten blank samples (without the target analyte) were tested, yielding an average current response of 44.044 μA with a standard deviation of 1.318 μA. According to the equation LOD = 3SD (where SD is the standard deviation of the blank samples), the limit of detection (LOD) for electrochemical detection was calculated to be 1.57 × 10^−11^ M.

Figure 9C displays the UV-Vis absorption spectra corresponding to different MAL concentrations. Within the concentration range of 10^−10^ M to 10^−5^ M, the absorbance of oxidized TMB (oxTMB) gradually increased with the rising concentration of MAL. As shown in Figure 9D, a good linear relationship was observed between the absorbance change (A − A_0_, where A is the measured absorbance and A_0_ is the blank) and the logarithm of MAL concentration. The baseline drift in Figure 9C was corrected by calculating the absorbance change value (A − A_0_); thus, it accurately reflects the signal variation induced by the change in target concentration. The absorbance intensities of spectra c–e (corresponding to concentrations of 10^−7^ to 10^−9^ M) are similar, which is because the amount of probe released exhibits an approximately linear competitive binding relationship with the target concentration in the low concentration range, resulting in a small difference in the amplitude of signal variation. The linear regression equation is:

A − A_0_ = 0.092LgC + 0.968 (2)

with a correlation coefficient of R^2^ = 0.992. As shown in Figure 10B, the average absorbance value of the blank samples was 0.1093, and the standard deviation was 0.00602. Consequently, the LOD for colorimetric detection was calculated to be 4.76 × 10^−11^ M.

The prepared electrochemical/colorimetric dual-mode aptasensor was compared with other previously reported methods for MAL detection. As shown in Table 1, compared with other methods, the approach proposed in this study exhibits a wider detection range and a lower LOD for MAL. These results indicate that this method offers certain advantages in the detection of MAL.

### 3.6. Specificity, Repeatability, and Stability of the Dual-Mode Aptasensor

Under optimized experimental conditions, the specificity, repeatability, and stability of the electrochemical/colorimetric dual-mode aptasensor were analyzed.

**Figure 11:**
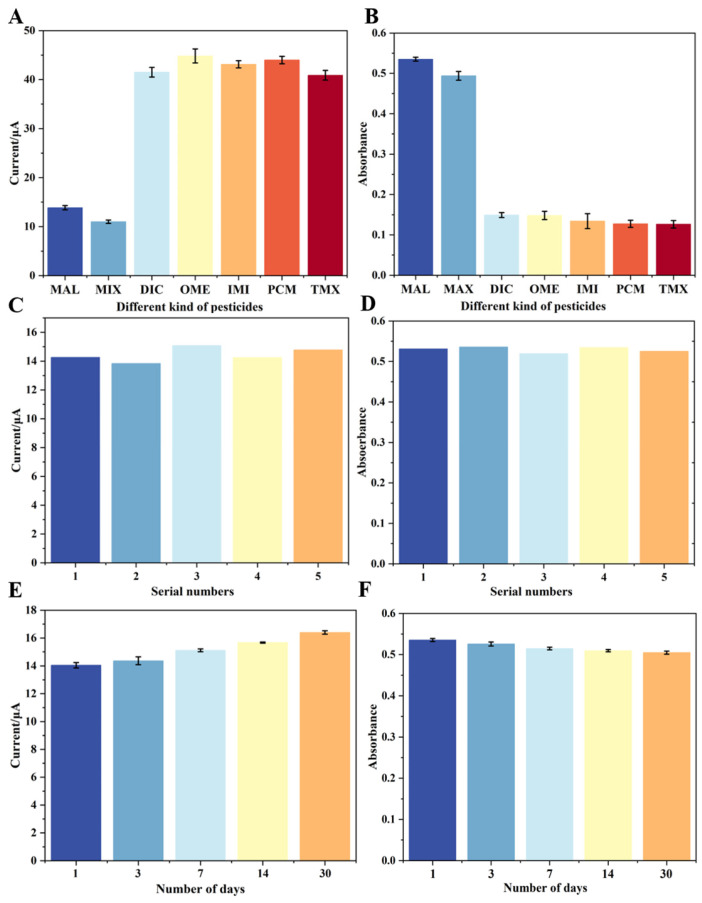
(**A**,**B**) Selectivity of the dual-mode aptasensor; (**C**,**D**) repeatability of the dual-mode aptasensor; (**E**,**F**) storage stability of the dual-mode aptasensor.

Five common pesticides were selected as potential interferents and grouped into seven sets: MAL, DIC, OME, IMI, PCM, TMX, and a mixture of all six pesticides (MIX). Each 1 μM pesticide sample was incubated under optimal conditions, followed by separation of the supernatant and precipitate, which were then subjected to electrochemical and colorimetric detection, respectively. As shown in Figure 11A, a significant decrease in DPV signal from the precipitate was observed only in the presence of the target analyte. Similarly, in Figure 11B, a high absorbance was only observed in the presence of MAL and in the MIX group. This is attributed to the strong specificity of the aptamer toward the target, which causes CuZr-MOF-cDNA to be competitively displaced into the supernatant. These results confirm that the sensor exhibits excellent specificity for MAL and is suitable for practical detection applications.

To demonstrate the repeatability of the fabricated sensor, five independent tests were performed under the same experimental conditions. As shown in Figure 11A,B, the results were highly consistent across replicates. The relative standard deviation (RSD) was 3.38% for electrochemical detection and 1.27% for colorimetric detection, indicating good repeatability.

The storage stability of the dual-mode sensor was evaluated, with detection performed on the supernatant and precipitate after 1, 3, 7, 14, and 30 days, using both electrochemical and colorimetric methods. As shown in Figure 11E,F, the RSDs were 4.33% for the colorimetric method and 2.39% for the electrochemical method, indicating good storage stability of the dual-mode sensor over a 30-day period.

### 3.7. Real Sample Analysis

Carrot and spinach were selected as representative vegetable samples. After sample pretreatment, MAL was spiked into the samples at concentrations of 100 nM, 10 nM, and 1 nM. The prepared dual-mode electrochemical/colorimetric aptasensor was then used for both electrochemical and colorimetric detection. The results are shown in Table 2. The recovery rates for electrochemical detection ranged from 87.71% to 100.71%, with RSD between 3.23% and 6.63% (The generally accepted criteria in the field of pesticide analysis: recovery rate 70–120%, RSD < 15%). For colorimetric detection, the recovery rates ranged from 93.24% to 107.68%, with RSDs between 7.53% and 10.75%.

These results indicate that the dual-mode electrochemical/colorimetric sensor exhibits good performance in detecting MAL in vegetable samples.

## 4. Conclusions

In this study, a dual-mode electrochemical/colorimetric aptasensor was developed by combining the peroxidase-like catalytic activity of bimetallic MOF materials with the magnetic Fe_3_O_4_@ZIF-8 nanocomposite materials. The sensor leverages the strong specificity of aptamers for target recognition and utilizes magnetic materials for colorimetric and electrochemical detection of the supernatant and precipitate. The dual-mode detection with electrochemical and colorimetric signals allows for mutual verification, enhancing the accuracy of the detection.

The fabricated dual-mode sensor has an electrochemical LOD of 1.57 × 10^−11^ M and a colorimetric detection LOD of 4.76 × 10^−11^ M, with a detection range from 1 × 10^−5^ to 1 × 10^−10^ M. When applied to actual sample testing, recovery rates of 87.71% to 100.71% (electrochemical) and 93.24% to 107.68% (colorimetric) were obtained. Verified results show that the proposed sensor exhibits excellent specificity, reproducibility, and stability. The mutual verification of electrochemical and colorimetric results makes the dual-mode aptasensor promising for precise detection of organophosphorus pesticides in vegetables.

## Figures and Tables

**Table 1:** Comparison of this study with other reported MAL detection methods.

Methods	Materials	Detection Range (M)	LOD (M)	Refs.
Colorimetric	paper-based	1.0 × 10^−4^–5.0 × 10^−2^	-	[16]
Electrochemical	SiC@CuO-NPs	3.0 × 10^−11^–3.0 × 10^−9^	1.0 × 10^−11^	[17]
Electrochemiluminescence	ZnO-Fe_2_O_3_	6.6 × 10^−11^–6.6 × 10^−7^	2.2 × 10^−11^	[18]
Electrochemical/colorimetric dual mode	Fe-MOF	3.0 × 10^−11^–1.5 × 10^−9^	1.4 × 10^−11^	[19]
Surface-enhanced Raman spectroscopy	AgNPs-PDMS	3.0 × 10^−7^–1.5 × 10^−5^	1.7 × 10^−7^	[20]
Fluorescence	SQDs@g-C_3_N_4_	1.0 × 10^−5^–1.2 × 10^−4^	2.0 × 10^−8^	[21]
Electrochemical/colorimetric dual mode	Fe_3_O_4_@ZIF-8-DNA@cDNA-CuZr-MOF	1.0 × 10^−10^–1.0 × 10^−5^	1.6 × 10^−11^	This work

**Table 2:** Determination of MAL in vegetable samples by dual-mode electrochemical/colorimetric sensors.

Methods	Sample	Added (nM)	Detection (nM)	RSD (%)	Recovery (%)
Electrochemical	Spinach	100	94.73	3.82	94.73
		10	9.12	4.07	91.23
		1	0.87	6.63	87.71
	Carrot	100	96.50	4.79	96.50
		10	10.07	6.44	100.71
		1	0.93	3.23	93.26
Colorimetric	Spinach	100	100.12	9.02	100.12
		10	9.54	7.53	95.43
		1	1.08	10.75	107.68
	Carrot	100	93.24	8.34	93.24
		10	9.89	7.76	98.87
		1	1.04	9.83	104.26

## Data Availability

The original contributions presented in this study are included in the article. Further inquiries can be directed to the corresponding author.

## Supplementary Materials

The following supporting information can be downloaded at: https://www.mdpi.com/article/10.3390/bios16020101/s1, Materials and Methods: Reagents and Instruments, Preparation of Nanomaterials, Preparation of Fe_3_O_4_@ZIF-8-DNA, Preparation of CuZr-MOF-cDNA, Fabrication of the Aptasensor, Electrochemical Detection, Sample Pretreatment.
