# Supplementary material for: Electrochemical/Colorimetric Dual-Mode Aptasensor Based on CuZr-MOF and Fe3O4@ZIF-8 for Detection of Malathion in Vegetables
Source: Biosensors (Basel). 2026 Feb 4;16(2):101. doi: 10.3390/bios16020101 (PMC12937831; doi:10.3390/bios16020101)
Supplement: Supplementary file 1 [file biosensors-16-00101-s001.zip › biosensors-4093413-supplementary.pdf]

1.Materials and Methods

1.1. Reagents and Instruments

Main reagents used in the experiment: phorate (PHT, 98%), omethoate (OME, 97.5%), and thiamethoxam (TMX, 98%) were purchased from Beijing Zhongke Quality Inspection Biotechnology Co., Ltd. (Beijing, China). Dichlorvos (DIC), imidacloprid (IMI, ≥97%), and procymidone (PCM) were obtained from Shanghai Aladdin Biochemical Technology Co., Ltd. (Shanghai, China). 1,4-benzenedicarboxylic acid (H_2BDC), zirconium tetrachloride ($ZrCl_4$), copper acetate ($Cu(CH_3COO)_2$), N,N-dimethylformamide (DMF), zinc nitrate hexahydrate ($Zn(NO_3)_2 \cdot 6H_2O$), 2-methylimidazole (2-MeIM), ferric chloride hexahydrate ($FeCl_3 \cdot 6H_2O$), sodium polystyrene sulfonate (PSS), and acetic acid were all analytical grade and obtained from Shanghai Macklin Biochemical Co., Ltd. (Shanghai, China). Dopamine was purchased from Shanghai Honyu Biotechnology Co., Ltd. (Shanghai, China). Potassium ferricyanide trihydrate ($K_4[Fe(CN)_6] \cdot 3H_2O$), potassium ferricyanide ($K_3[Fe(CN)_6]$), and potassium chloride (KCl) were all purchased from Shanghai Macklin Biochemical Co., Ltd. Phosphate-buffered solution (PBS) and bovine serum albumin (BSA) were purchased from Sinopharm Chemical Reagent Co., Ltd. (Shanghai, China). The broad-spectrum OPs aptamer sequences were synthesized by Sangon Biotech Co., Ltd. (Shanghai, China), The aptamer DNA sequence used in this study was 5’-COOH-ATCCGTCACACCTGCTCTTATACACAATTGTTTTTCTCTTAACTTCTTGACTGCTGGTGTTGGCTCCCGTAT-3’, and the complementary cDNA sequence was 5’-NH_2-$(CH_2)_6$-GGGAGCCAACACCAG-3’[11]. All reagents used in the study were of analytical grade.

All electrochemical measurements were performed using a standard three-electrode system consisting of a saturated calomel electrode (SCE) as the reference electrode, a platinum electrode as the counter electrode, and a bare or modified glassy carbon electrode (GCE) as the working electrode. Measurements were carried out in 5

mM $[Fe(CN)_6]^{3-/4-}$ solution containing 0.1 M KCl as the supporting electrolyte. The instruments used in this study included an electrochemical workstation (CHI 660D, Shanghai Chenhua Instruments Co., Ltd., China), a UV–visible spectrophotometer (UV-2550, Shimadzu, Japan), a scanning electron microscope (JSM-7800, JEOL Ltd., Japan), a Fourier-transform infrared (FTIR) spectrometer (Nicolet iS50, Thermo Fisher Scientific, USA), an X-ray powder diffractometer (D8 ADVANCE, Bruker Corporation, Germany), a high-speed centrifuge (Sorvall ST 16R, Thermo Fisher Scientific, USA), an analytical balance (AL104, Shanghai Mettler Toledo Instrument Co., Ltd., China), micropipettes (Eppendorf Research plus, Eppendorf AG, Germany), and an ultrasonic cleaner (KQ3200E, Kunshan Ultrasonic Instruments Co., China).

1.2. Preparation of Nanomaterials

The CuZr-MOF was synthesized following a procedure developed in our previous work[12]. Specifically, 8.5 mM of 1,4-benzenedicarboxylic acid (H_2BDC) was dissolved in 20 mL of N, N-dimethylformamide (DMF) under stirring at room temperature until completely dissolved. Subsequently, a mixed solution of 5 mM zirconium tetrachloride ($ZrCl_4$) and 2 mM copper(II) acetate $[Cu(CH_3COO)_2]$ was added dropwise to the H_2BDC solution under continuous stirring. The resulting mixture was stirred for 6 h at room temperature, during which a white precipitate was formed. The precipitate was collected by centrifugation at 12000 rpm for 30 min and washed sequentially with DMF and ethanol to remove any unreacted residues. The purified product was then redispersed in 20 mL of acetone, and 1 mL of acetic acid was added to maintain an acidic environment. The resulting mixture was transferred to a Teflon-lined autoclave and heated at 120 °C for 24 h. After cooling to room temperature, the product was again collected by centrifugation, thoroughly washed with ultrapure water and ethanol, and finally dried under vacuum at 60 °C for 12 h.

The synthesis of ZIF-8 was based on previously reported methods [13]. Briefly, 1.17 g of zinc nitrate hexahydrate ($Zn(NO_3)_2 \cdot 6H_2O$) was dissolved in 8 g of deionized water to form the zinc salt solution. Simultaneously, 22.70 g of 2-methylimidazole

was dissolved in 80 g of deionized water to prepare the ligand solution. The zinc salt solution was then slowly poured into the ligand solution under continuous stirring at room temperature. After stirring for approximately 5 min, a white precipitate was obtained by centrifugation. The precipitate was washed multiple times with deionized water to remove unreacted reagents or by-products, and subsequently dried in an oven at 60 °C. The final yield of ZIF-8 was approximately 80%.

Fe_3O_4 nanospheres were synthesized via a solvothermal method [14]. First, 2.7 g of $FeCl_3·6H_2O$ was dissolved in 50 mL of ethylene glycol under magnetic stirring. After stirring for 30 min, a clear yellow solution was obtained. Then, 5.75 g of sodium acetate (CH_3COONa) was added to the solution. After an additional 30 min of stirring, the mixture was transferred into a Teflon-lined stainless steel autoclave and heated at 200°C for 8 h. After cooling to room temperature, the black magnetic nanospheres were collected using a magnet and washed several times with ethanol.

Fe_3O_4@ZIF-8 nanocomposites were synthesized according to methods from the literature [15]. First, surface modification of Fe_3O_4 nanoparticles was carried out using sodium polystyrene sulfonate (PSS) as a surfactant to impart a negative surface charge, enabling the adsorption of positively charged metal ions and promoting ZIF-8 nucleation. Specifically, 0.01 g of Fe_3O_4 nanoparticles was dispersed in 30 mL of 0.3% (w/v) PSS aqueous solution. The suspension was ultrasonicated for 20 min and stirred to ensure homogeneous dispersion. The magnetic particles were then collected using an external magnetic field and washed three times with distilled water to remove excess PSS. The collected Fe_3O_4 particles were redispersed in 30 mL of methanol, followed by the addition of 0.225 g of $Zn(NO_3)_2·6H_2O$ and 0.622 g of 2-methylimidazole. The mixture was stirred at 50 °C for 3 h to promote the growth of the ZIF-8 shell. After the reaction, the product was collected via magnetic separation, washed several times with ethanol, and vacuum-dried at 60 °C for 12 h to obtain the final Fe_3O_4@ZIF-8 composite material.

1.3. Preparation of Fe_3O_4@ZIF-8-DNA

To immobilize the carboxyl-modified aptamer, the surface of the synthesized

Fe_3O_4@ZIF-8 nanocomposite material was first aminated using a polydopamine coating method [16]. Fe_3O_4@ZIF-8 nanocomposites were dispersed in Tris buffer (pH 8.5), and dopamine (2 mg/mL) was added. The mixture was stirred at room temperature for 24 h, allowing the formation of a polydopamine (PDA) coating rich in amine groups on the surface. After centrifugation and washing, the composite was redispersed in PBS buffer (pH 7.4) and sonicated for 30 min to ensure uniform dispersion.

A total of 1 mL of the aminated Fe_3O_4@ZIF-8 solution was then mixed with 10 μL of 1 μM carboxyl-modified DNA probe. EDC and NHS (in a 4:1 molar ratio) were added to activate the carboxyl groups. The mixture was incubated at room temperature for 2 h, allowing the DNA probe to covalently bind to the Fe_3O_4@ZIF-8 surface via the reaction between carboxyl and amine groups. To block non-specific binding sites, 1% BSA solution was added and incubated at room temperature for 2 h [17]. The final Fe_3O_4@ZIF-8-DNA composite was then dispersed in PBS buffer and stored at 4 °C for later use.

1.4. Preparation of CuZr-MOF-cDNA

CuZr-MOF nanomaterials were dispersed in PBS buffer (pH 7.4), and 1 mL of the solution was mixed with 10 μL of 1 μM cDNA. EDC and NHS (in a 4:1 molar ratio) were added to activate the carboxyl groups on the CuZr-MOF surface. The mixture was incubated at room temperature for 2 h, allowing the cDNA to bind covalently to the CuZr-MOF surface via amide bond formation between the carboxyl and amine groups. After blocking non-specific binding sites with 1% BSA, the CuZr-MOF-cDNA composite was dispersed in PBS buffer and stored at 4 °C for later use.

1.5. Fabrication of the Aptasensor

A total of 20 μL of the prepared Fe_3O_4@ZIF-8-DNA was mixed with an equal volume (1:1 ratio) of CuZr-MOF-cDNA and incubated at room temperature for 1 h. During this time, the aptamer DNA hybridized with the complementary cDNA, stabilizing the binding of CuZr-MOF-cDNA to Fe_3O_4@ZIF-8-DNA. Subsequently, 10

μL of the target solution was added and incubated for 1 h. In the presence of the target, the aptamer specifically binds to the target, causing the cDNA to dissociate from the DNA, thereby releasing CuZr-MOF-cDNA into the supernatant. After the target-induced release, magnetic separation was used to quickly separate the target-captured composite from the solution. Both the supernatant and precipitate were collected.

The free CuZr-MOF-cDNA in the supernatant participated in a TMB catalytic reaction, generating a blue product. A total of 5 μL of the collected supernatant was mixed with 20 μL of TMB solution (1 mM), 10 μL of 1% H_2O_2 solution, and 15 μL of sodium acetate buffer (pH 5.0). The mixture was incubated at 25 °C for 20 min. The colorimetric signal intensity was then measured at 652 nm using a UV-Vis spectrophotometer.

For the precipitate part, the target-captured Fe_3O_4@ZIF-8-DNA and un-released CuZr-MOF-cDNA were used for electrochemical testing. After washing the precipitate with deionized water, it was redispersed in PBS buffer (pH 7.4), and 5 μL was dropped onto a pre-treated glassy carbon electrode (GCE). After drying, electrochemical signals were tested in 5 mM potassium ferrocyanide solution ($[Fe(CN)_6]^{3-/4-}$).

1.6. Electrochemical Detection

Electrochemical analyses, including cyclic voltammetry (CV), differential pulse voltammetry (DPV), and electrochemical impedance spectroscopy (EIS), were conducted to evaluate the performance of the aptasensor. These experiments were performed using an electrolyte solution consisting of 0.1 M potassium chloride (KCl), 5 mM potassium ferricyanide ($K_3[Fe(CN)_6]$), and 5 mM potassium ferrocyanide ($K_4[Fe(CN)_6]$). CV measurements were carried out at a scan rate of 100 mV/s over a potential range from -0.2 V to 0.6 V. DPV was performed over the same potential range, utilizing a step potential of 4 mV, a pulse amplitude of 50 mV, and a pulse duration of 0.2 seconds. Simultaneously, EIS measurements were conducted over a frequency range from 0.1 Hz to 100000 Hz, with an oscillation amplitude of 5 mV.

1.7. Sample Pretreatment

Carrot and spinach were selected as vegetable samples. The pretreatment method for the vegetable samples is similar to the one described in Section 2.2.6. The carrot and spinach samples were purchased from the Da Run Fa supermarket in Zibo, Shandong Province. After cleaning, the samples were cut into 1-2 mm sized pieces. A total of 5 g of the chopped vegetable samples were weighed and placed into centrifuge tubes. Then, standard MAL solutions with concentrations of 1 nM, 10 nM, and 100 nM were added to each tube, followed by standing for 24 h. Next, 10 mL of acetonitrile was added to each tube, and the mixture was vigorously shaken for 1 hour to ensure thorough mixing. The mixture was then centrifuged at 10,000 rpm for 10 min. Afterward, the solution was filtered using a 0.22 μm filter membrane to remove suspended particles. The filtered solution was added to the prepared Fe_3O_4@ZIF-8-DNA@cDNA-CuZr-MOF system and incubated. Magnetic separation was performed for dual-mode detection. The measured values were then applied to the established standard curve to calculate the detection concentration.
